# Employing the Interpretable Ensemble Learning Approach to Predict the Bandgaps of the Halide Perovskites

**DOI:** 10.3390/ma17112686

**Published:** 2024-06-02

**Authors:** Chao Ren, Yiyuan Wu, Jijun Zou, Bowen Cai

**Affiliations:** 1 Jiangxi Province Key Laboratory of Nuclear Physics and Technology, East China University of Technology, Nanchang 330013, China; 2021110241@ecut.edu.cn (C.R.);; 2 Engineering Research Center of Nuclear Technology Application, East China Institute of Technology, Ministry of Education, Nanchang 330013, China; 3 Jiangxi Engineering Laboratory on Radioactive Geoscience and Big Data Technology, East China University of Technology, Nanchang 330013, China; 4 School of Information Engineering, East China University of Technology, Nanchang 330013, China; 5 School of Nuclear Science and Engineering, East China University of Technology, Nanchang 330013, China

**Keywords:** machine learning, halide perovskite, Shapley additive explanations, bandgap, regression

## Abstract

Halide perovskite materials have broad prospects for applications in various fields such as solar cells, LED devices, photodetectors, fluorescence labeling, bioimaging, and photocatalysis due to their bandgap characteristics. This study compiled experimental data from the published literature and utilized the excellent predictive capabilities, low overfitting risk, and strong robustness of ensemble learning models to analyze the bandgaps of halide perovskite compounds. The results demonstrate the effectiveness of ensemble learning decision tree models, especially the gradient boosting decision tree model, with a root mean square error of 0.090 eV, a mean absolute error of 0.053 eV, and a determination coefficient of 93.11%. Research on data related to ratios calculated through element molar quantity normalization indicates significant influences of ions at the X and B positions on the bandgap. Additionally, doping with iodine atoms can effectively reduce the intrinsic bandgap, while hybridization of the s and p orbitals of tin atoms can also decrease the bandgap. The accuracy of the model is validated by predicting the bandgap of the photovoltaic material MASn_1−x_Pb_x_I_3_. In conclusion, this study emphasizes the positive impact of machine learning on material development, especially in predicting the bandgaps of halide perovskite compounds, where ensemble learning methods demonstrate significant advantages.

## 1. Introduction

**Figure 1:**
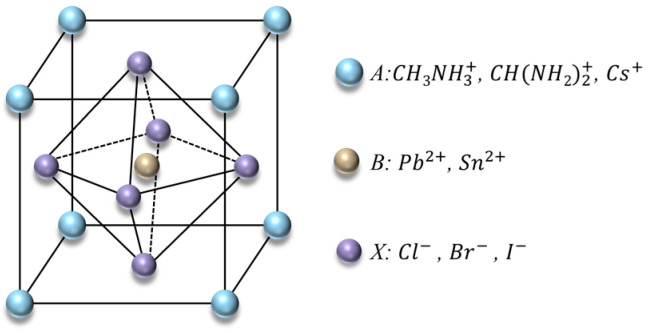
Crystal structure of the halide perovskites [3].

Halide perovskite is a material with a ABX_3_ type distinctive crystal lattice structure. as shown in Figure 1. A, B, and X represent cations, metal cations, and halogen anions respectively. Generally, the cation of A part is formamidinium (${FA}^{+}{\left(({CH}_{3}{NH}_{3}\right)}^{+})$), methylammonium (${MA}^{+}(CH{\left({NH}_{2}\right)}_{2}^{+})$) and cesium (${Cs}^{+}$), while the metal cations are lead (${Pb}^{2+}$) or tin (${Sn}^{2+}$), and the halide anions are chlorine (${Cl}^{-}$), bromine (${Br}^{-}$), or iodine ($I^{-}$). In this crystal structure, the B ion is located at the center of the cubic unit cell, the X ion is located at the center of the octahedron, and the A ion is located at the vertex of the cubic unit cell [1,2]. For example, methylammonium lead halide (MALHs) is a typical halide perovskite compound with the chemical formula CH_3_NH_3_PbX_3_. In this formula, ${\left({CH}_{3}{NH}_{3}\right)}^{+}$ denotes the A cation, Pb^2+^ denotes the B cation, and X denotes the chlorine (${Cl}^{-}$), bromine (${Br}^{-}$), or iodine ($I^{-}$). The stability and unique electrical properties of this structure confer significant practical applications in areas such as photovoltaics and optoelectronic devices. In recent years, due to a series of excellent photoelectric properties, perovskites have shown excellent application prospects in the fields of light-emitting diodes, lasers, photodetectors, solar cells, and memory.

However, perovskite was previously less noticed due to its instability and low photovoltaic conversion efficiency. It was not until after 2009 that the efficiency of perovskite solar cells increased significantly, rising from 3.8% [4] to 25.2% [5]. Due to rapid efficiency advancements, ABX_3_ halide perovskite materials have emerged as foundational materials for solar cells and optoelectronic devices, garnering widespread attention. Perovskites, as direct bandgap semiconductor materials, exhibit excellent bandgap tunability. Bandgap tunability implies that ABX_3_-type perovskites can adjust their bandgap by altering their elemental composition, enabling better spectral responsiveness within a certain range. Since the energy conversion efficiency of solar cells depends on the wavelength range of sunlight that can be absorbed and the energy distribution of photons in sunlight, and the material’s energy gap determines the wavelength of photons that can be absorbed, selecting the appropriate energy gap is crucial. This allows for absorbing the band with the highest energy proportion of sunlight, thereby maximizing energy conversion efficiency. Consequently, the design of the perovskite layer’s bandgap directly influences the spectral response range of solar cells and their photoelectric conversion efficiency [6,7,8].

However, accurately and efficiently calculating the bandgap of perovskite, regulated by different elements, remains a significant challenge. Currently, most calculations rely on the Kohn–Sham bandgap obtained from density functional theory (DFT) as an approximate measure [9]. However, the question at this stage is how to calculate the electronic and optical properties of semiconductor materials with reasonable accuracy based on a variety of DFT-based calculations, including the local density approximation (LDA), LDA-1/2, the modified Baker–Johnson (mBJ) method, and the generalized function of the Hay–Szekhov method (HSE06) [10,11]. For an extended period, software like the Vienna Ab-initio Simulation Package and Material Studio (CASTEP toolkit) have been widely utilized for theoretical calculations of crystal materials’ properties. However, despite their potency, the high computational complexity, intricate reaction mechanisms, demanding hardware prerequisites, sluggish calculation speed, and substantial resource consumption render material calculations costly and inefficient. Moreover, the intricate composition of perovskite solar cells (PSCs) exacerbates the challenge of accurately determining their bandgap, constrained by discrepancies between software-calculated values and experimental results [12].

Over the past few years, artificial intelligence has experienced rapid development, injecting newfound vigor into numerous research fields. The application of data science and information theory approaches has yielded significant success in diverse areas, including chemical informatics, game theory, pattern recognition, artificial intelligence, and event prediction, with current widespread utilization in materials informatics [13,14,15]. Machine learning (ML), a proven and effective approach for predicting the physical and chemical properties of materials [16,17], is extensively employed in the semiconductor materials domain. Ayana Ghosh et al., for instance, established a connection between actinide magnetism and structure by assembling and mining two datasets. They utilized a regression algorithm to discern the accessible attributes of the material system, achieving a root mean square error of 0.17 μB and 0.19 μB for predicting spin moments and orbital moments, respectively. The random forest classification algorithm demonstrated a 76% accuracy in predicting such systems [17]. Vladislav Gladkikh et al. investigated the correlation between bandgap and elemental properties in ABX_3_ perovskite materials using machine learning, highlighting Kernel Ridge Regression and Extremely Randomized Trees as top-performing models. Additionally, Alternating Conditional Expectation (ACE) is explored, offering graphical interpretability, while Support Vector Machines are employed for metallic vs. non-metallic perovskite classification [18]. David O. Obada et al. utilized explainable machine learning algorithms to predict ABX_3_ type perovskite bandgaps and identified key features, such as Pauling electronegativity, impacting predictions. CatBoost and XGBoost models demonstrated superior performance, highlighting the potential of machine learning for rapidly and accurately predicting material properties, particularly beneficial for engineering [19]. Steven K. Kauwe et al. showed how ensemble learning methods can combine experimental data to effectively model experimental data, with the root mean square error of bandgap prediction reduced by more than 9% [20]. Researchers have selected different ML approaches to construct specific bandgap prediction models based on their research systems [21].

Our work focuses on using an ensemble learning-based ML approach to explore which types of models are most effective for predicting bandgaps. We centered our experiments on halide perovskite bandgap prediction and analyzed the results from multiple dimensions, including nonlinearity, kernel functions, and ensemble algorithms. One notable advantage of this work is the utilization of ensemble learning models, which exhibit superior performance when contrasted with traditional machine learning models. By delineating the variances among different models, the study offers valuable insights for prioritizing the prediction of bandgaps for similar materials. Additionally, beyond presenting predictive outcomes, the research delves into the primary factors influencing perovskite bandgaps based on existing data. It employs Shapley Additive exPlanations (SHAP), an interpretable machine learning framework, to elucidate these findings. Moreover, with the incorporation of inorganic A-site elements into halide perovskite systems [22], this work has contributed to the advancement of binary systems and ternary cationic perovskites, including MA, FA, and Cs, aiming to enhance the quality and stability of perovskite materials.

## 2. ML Approach

### 2.1. Experimental Environment

The machine learning program in this experiment operates within the Python 3.7 environment, primarily leveraging the Scikit-learn library. Scikit-learn not only furnishes a multitude of machine learning algorithms and models but also provides an array of tools for data preprocessing, and model fitting, selection, and evaluation, alongside numerous other practical utilities. It finds extensive application in tasks encompassing prediction, classification, clustering, and dimensionality reduction. The experimentation was conducted on a Windows 11 (64-bit) platform, featuring an AMD Ryzen 7-5800H 8-core processor operating at a frequency of 3.20 GHz, complemented by Radeon Graphics with 2G. The Jupyter Notebook interactive application served as the primary interface for the experiment. Various models sourced from Python libraries such as sklearn (v1.0.2), xgboost (v1.6.1), lightgbm (v3.3.2), among others, were employed.

**Figure 2:**
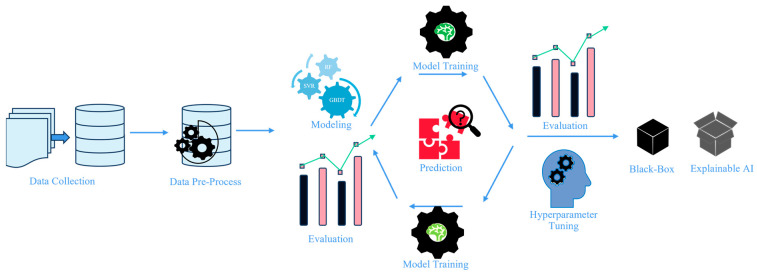
Interpretable machine learning approach workflow.

The general machine learning process encompasses data acquisition, data extraction, feature selection, model training, tuning optimization, model evaluation, and regression (prediction)/classification. The workflow diagram illustrating the interpretable machine learning approach in Figure 2.

### 2.2. ML Model

In this study, we employed several models, including Support Vector Regressor (SVR) [23], Kernel Ridge Regression (KRR), Random Forest (RF) [24], Gradient Boosting Decision Tree (GBDT), Light Gradient Boosting Machine (LightGBM) [25], and Extreme Gradient Boosting Decision Tree (Xgboost). Both RF and GBDT, as well as LightGBM and Xgboost, are ensemble models based on decision trees in machine learning. Ensemble learning, a meta-approach that combines weak learners into an optimized model, was utilized to improve overall performance. Due to their composition of multiple weak learners, ensemble models have higher accuracy than general ML models, better reduction of overfitting, and prevention of accuracy decline during testing, despite good performance during training.

**Figure 3:**
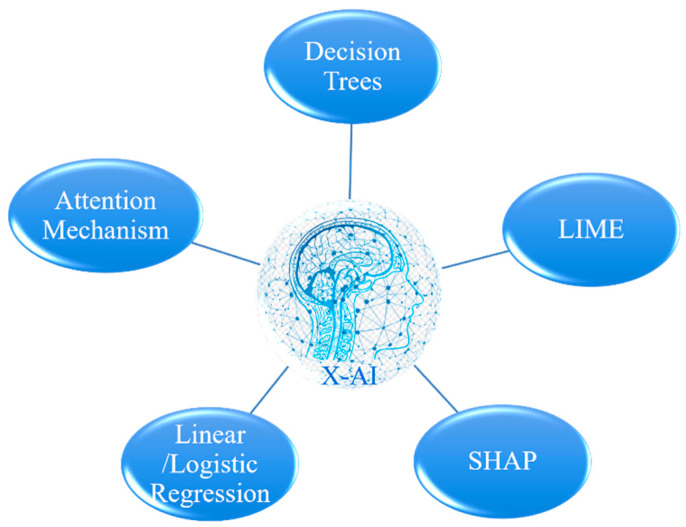
A few important interpretable AI approaches.

Additionally, Explainable Artificial Intelligence (X-AI) techniques are increasingly being integrated into materials development to enhance the transparency and comprehensibility of machine learning model decision-making. This domain involves creating algorithms that are inherently interpretable and developing methods to elucidate existing models. A few important interpretable AI approaches is shown in Figure 3. (1) Decision trees, which illustrate decision-making through a tree structure, are easily understood and interpretable, and visually represent the decision process. However, they are prone to overfitting with extensive tree depth and handling continuous variables can be complex. (2) Linear Regression, which models variable relationships using linear equations, and Logistic Regression, suitable for regression problems. The former is ideal for regression, the latter for classification; both models are simple and diverse, but struggle with explaining nonlinear relationships. (3) Local Interpretable Model-agnostic Explanations (LIME), which approximates the original model by generating data around it and training a new, simple model on these data. (4) SHAP (SHapley Additive exPlanations), offering global and local explanations based on Shapley values from cooperative game theory to assign feature contributions to model output, applicable to any machine learning model but computationally intensive for models with many features. (5) Attention mechanism in interpretable neural networks, enhancing deep learning model interpretability without compromising performance, though interpretations may not be as intuitive or reliable as simpler models. The goal of interpretable AI development is to reconcile performance and interpretability, allowing complex machine learning models to maintain high performance while ensuring their decision-making is understandable and trusted by humans. Each method has strengths and limitations, and the choice depends on specific application scenarios, model complexity, and interpretative requirements.

SHAP is based on the Shapley value in game theory, measuring each cooperator’s contribution to the gain. In supervised learning, SHAP breaks down the model’s impact on each feature’s prediction, offering clarity on feature importance. By evaluating all feature combinations, it shows how each feature value affects the output. In our research, we apply SHAP to interpret our ensemble learning models, particularly the gradient boosting decision tree model. It quantifies the contribution of each feature to the prediction made by the model, providing insight into the physical reasons underlying the results of machine learning. This helps us understand how different features influence ABX_3_ perovskite bandgap prediction, enhancing interpretability [26].

### 2.3. Dataset

The dataset utilized in this study comprises 245 experimental data of halide bandgaps, collected from a plethora of academic articles, including renowned publishing platforms such as ELSEVIER, Wiley Online Library, MDPI, and ACS, up until June 2023 (see Supplementary Materials). This dataset is integrated with the molecular formulae of perovskite materials with different doping ratios (different atomic occupancies) as features, and bandgap values measured during the experiments as targets. The molecular structures of these compounds are extensively documented in the literature, readily accessible, and considered reliable, which constituted the primary motivation for their selection in constructing the dataset. Prior to commencing experiments, all data underwent meticulous inspection and screening. Acknowledging the inherent errors stemming from diverse experimental conditions, we retained authentic data while excluding duplicates, culminating in a final dataset comprising 245 sets of experimental data. Given the relatively small size of the original dataset, we partitioned the perovskite data into training (220 sets) and testing (25 sets) sets at a ratio of 9:1 to ensure adequate model training during experimentation. It is worth emphasizing that during the data cleaning process, discrepancies were encountered in the proportions of Br, I, and Cl atoms within the halide data sets. Consequently, normalization was applied to all data, compressing the ratios by a factor of three, thereby constraining the atomic ratios within the 0–1 range. This measure aims to mitigate experimental errors induced by numerical disparities.

### 2.4. Features Selection

**Figure 4:**
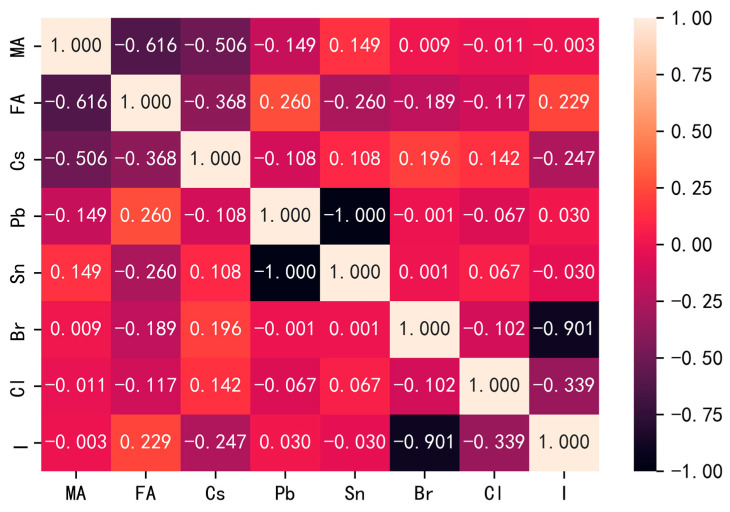
Heat map of characteristic coefficients.

Feature selection is a pivotal step in machine learning, as an excess of irrelevant features within a dataset can augment model complexity. The employment of feature filtering serves to diminish the number of features, enhancing model accuracy while reducing the time overhead through the elimination of irrelevant or redundant features. Conversely, the curation of genuinely pertinent features streamlines the model and aids in comprehending the data generation process. For feature filtering, we utilized the Pearson correlation coefficient formula to compute the correlation coefficient between each feature (refer to the Supplementary Material for the calculation formula). Typically ranging from −1.0 to 1.0, the Pearson correlation coefficient serves as a quantitative measure. Figure 4 visually represents the heatmap generated by Pearson correlation coefficients among eight features, while Figure S1 (refer to the Supplementary Materials) illustrates the scatter plot calculated using Pearson coefficients for various properties.

From Figure 4 and Figure S1, we can believe that there is a moderate negative correlation between MA and FA cations at the A site, and a weak linear relationship between A and B site cations; in other words, the linear relationship between MA and FA is stronger than that of Cs, and there is no large linear correlation between A and B sites. The B-position cations, on the other hand, show a certain degree of linear relationship due to the difference in the number of atoms in the center of Pb and Sn. In addition, due to the low proportion of Cl atoms in the dataset, which leads to the presence of Br and I atoms, the X atoms show a certain degree of linear relationship. This conclusion agrees with the first-principles calculations. In ABX_3_ halide perovskites, X- and B-site atoms significantly contribute near-gap electronic states, while A-site atoms exhibit negligible contributions. This is attributed to the strong hybridization between B-site s orbitals and X-site p orbitals in the valence bands, leading to the formation of conduction bands through antibonding interactions [27]. Furthermore, the coupling between Pb atom s and p orbitals contributes to the creation of conduction bands, given the ion characteristics of ABX_3_ perovskites. Consequently, the bonding interactions between valence band and conduction band states are minimal, particularly regarding Br atom contributions to conduction bands. Therefore, bonding interactions between valence band and conduction band states are negligible towards Br atom contributions in conduction bands. However, despite the fixed bandgap of ABX_3_ perovskites, variations occur due to changes in chemical composition. Considering the relatively small number of features in our dataset and the absence of linear correlation among A, B, and X site variables, we opted to retain all dataset features, with bandgap values serving as the target features.

Evaluation metrics play a crucial role in assessing the accuracy of machine learning models. The comparison between the predicted values and actual values provides an intuitive understanding of the model’s capability to accurately predict our target bandgap. In our experimental analysis, we employed mean absolute error (MAE), root mean squared error (RMSE), and R-squared (R^2^) as evaluation benchmarks for the model. Unlike the Pearson correlation coefficient, R^2^ serves as a determining factor in elucidating how independent variables influence dependent variables. Generally, a higher R^2^ value indicates better explanatory power for the independent variables in regression analysis, closely fitting the data. Moreover, given the limitation of training data, we adopted a 6-fold cross-validation method during model training. The average score across all models was then calculated to ensure a robust evaluation process.

### 2.5. Hyperparameters

The training dataset underwent model training, aiming to achieve optimal performance. To mitigate potential overfitting, a 6-fold cross-validation strategy was implemented alongside grid search or Bayesian optimization to fine-tune hyperparameters. Eventually, a model exhibiting the highest relative accuracy on the training dataset was derived. Given that the SVR model has multiple kernel functions such as linear, polynomial, radial basis function (RBF), and sigmoid kernels, we utilized grid search to select the kernel function that best predicts bandgaps. The search results indicated that RBF kernel function had the highest accuracy during training. Similarly, grid search was utilized for GBDT, XGBoost, KRR, and RF models to identify the optimal hyperparameters. For LightGBM model, we adopted Bayesian optimization for hyperparameter tuning. Table 1 presents the optimal hyper-parameters for each model. Subsequently, the test dataset was applied to all six models, and predictions were made accordingly.

## 3. Results and Discussion

**Figure 5:**
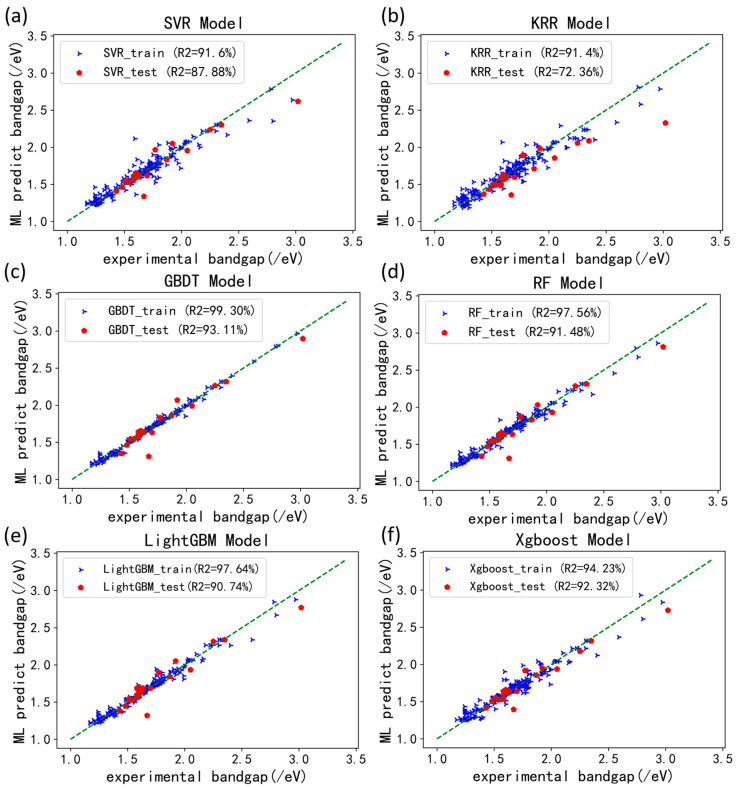
Correlation between predicted and experimental values of different models for the dataset (/eV). (**a**) Prediction result of SVR model; (**b**) prediction result of KRR model; (**c**) prediction result of GBDT model; (**d**) prediction result of RF model; (**e**) prediction result of LightGBM model; (**f**) prediction result of Xgbooost model.

Upon importing the training and testing datasets into the model, Figure 5 illustrates correlation plots of predicted and experimental values for various models. In the graph, blue triangles represent predicted results on the training dataset, while red pentagons depict predictions on the testing dataset. The dashed line serves as the benchmark.

Notably, SVR (Figure 5a) and KRR (Figure 5b) regression models exhibit scattered testing predictions, particularly in the bandgap range of 1.7–2.4 eV, where deviations are notably large, despite satisfactory training results. Specifically, while the SVR model’s predictions align better with experimental data than the KRR model overall, it tends to underestimate bandgap values on the training set as a whole, especially when exceeding 2.5 eV. It is apparent that the precision of these two models is not high. In contrast, GBDT (Figure 5c), RF regression model (Figure 5d), LightGBM (Figure 5e), and XGBoost (Figure 5f) demonstrate highly accurate training predictions that closely match experimental data, surpassing SVR and KRR algorithms in terms of accuracy. However, Figure 5e highlights that while LightGBM delivers relatively favorable results on the training set, it exhibits significant fluctuations in bandgap predictions within the range of 1.55 to 1.95 eV, potentially indicating an overfitting.

After conducting these analyses, we proceeded to compare the RMSE, MAE, and R^2^ values for all models in both the training and test sets. The data presented in Table 2 indicate that the error scores for the training set are universally higher than those for the test set. Notably, the KRR model stands out as it performs less optimally, exhibiting an R^2^ of 91.4% in the training set, but only achieving an R^2^ value of 72.36% in the test set, implying a potential occurrence of overfitting. In contrast, the performance of several other models in both the training and test sets displayed minimal differences, signifying an absence of overfitting. Among these, the GBDT model in the test set stands out with an RMSE of 0.090 eV and an R^2^ value of 93.11%, showcasing the highest accuracy among all regression models. The excellence of the GBDT model can be attributed to its superior fitting results in comparison to other models, resulting in remarkable predictive accuracy. Similarly, other ensemble learning models such as XGBoost, RF, and LightGBM also exhibit satisfactory prediction precision, boasting RMSE values of 0.095 eV, 0.100 eV, and 0.104 eV respectively. This underscores the effectiveness of the ensemble learning-based approach in accurately predicting the bandgap of perovskite materials and offering valuable guidance for studying the bandgaps of analogous materials.

Results from Table 2 unequivocally demonstrate that ensemble learning models surpass conventional ones in predicting bandgaps in halide perovskites, owing to their optimized algorithmic loss functions. However, the complexity of a prediction model does not always guarantee superior performance, as indicated by both RMSE and MAE values. Notably, experimental findings indicate that the GBDT model excels in accuracy compared to XGBoost and LightGBM. Despite GBDT’s utilization of a loss function employing solely negative gradient expansion on error segments, while XGBoost employs second-order Taylor expansion on error components, the optimization gains are not significantly heightened and may even result in lower predictive precision compared to GBDT. LightGBM’s advantage lies in its capacity to parallelize model computation while maintaining training accuracy, leading to shorter training times. Although LightGBM’s RMSE slightly underperforms compared to GBDT in the results, we attribute this to the dataset’s limited size, suggesting that the model’s performance might not be fully realized. Based on the training results from the available data, ensemble learning-based decision tree models like GBDT, XGBoost, and LightGBM exhibit prediction accuracies exceeding 90% for halide perovskite bandgaps.

**Figure 6:**
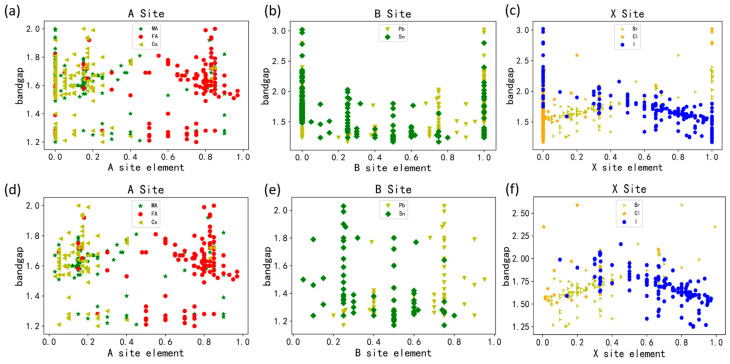
Correlation of the characteristic elemental ratios and bandgaps in the crystal structure in the A, B, and X site. Includes A site (MA, FA, Cs), B site (Pb, Sn), and X site (Br, Cl, I), (**a**–**c**) before processing and (**d**–**f**) after processing.

To investigate the potential relationship between the components of halide perovskites and their bandgap, we conducted an analysis of the atomic element ratios at A, B, and X sites alongside the distribution of bandgaps. As depicted in Figure 6, a comparison between datasets (a–c) and (d–f) reveals that the presence of only one type of atom at A, B, or X sites correlates with an overall increase in the bandgap width of perovskites. Consequently, we excluded experimental data featuring a single atom ratio for further analysis. From Figure 6a,d, it is evident that the distribution of three different types of atoms at the A site is more scattered, exerting a relatively minor influence on the variation of bandgap distribution. Figure 6b,e highlight that a high ratio of Sn atoms diminishes the bandgap width of perovskites. This phenomenon can be attributed to the pronounced spin–orbit coupling (SOC) between heavy metals such as Sn and Pb, coupled with the low-energy electronic structure inherent in halide perovskites [28]. Specifically, the alloying of heavier Pb atoms with lighter Sn atoms leads to an enhanced spin–orbit coupling (SOC) due to their contributions from s orbitals to hybridized states. This arises from the disparity in energy levels of the Pb s orbitals, which are positioned at lower energy levels in valence band states compared to Sn s orbitals, owing to differences in the non-hybridized s orbital energy levels for each atom. Consequently, there exists a notable contrast in their contributions to SOC, as Hu et al. summarized [28,29]. For most A-site and x-site alloys, the band gap of the alloys lies between the two band gaps of the original compositions while the bending is relatively small; meanwhile, b-site alloys such as Pb-Sn have higher bending and the band gap of the alloys is even lower than that of the two compositions, mainly due to the fact that perovskite’s band structure strongly depends on the b-site atoms. For example, in APbX_3_, lead-filled 6s orbitals and empty 6p orbitals determine the valence band maximum and the conduction band minimum. This observation also explains our inference [30,31].

From Figure 6c,f, it is evident that an increase in the proportion of iodine atoms correlates with a decrease in the bandgap of perovskite, akin to the effect observed by Volonakis et al. utilizing low bandgap materials such as iodides or alternative noble metals [32]. The transition from chlorine to iodine as double halides induces a significant reduction in the predicted fundamental bandgap. This characteristic enables iodides like methylammonium lead iodide to achieve an optimal bandgap range in single-junction photovoltaic cells. Conversely, augmenting the proportion of bromine atoms elevates the bandgap value owing to variances in atomic radii and bonding lengths. For instance, in CsSnI_3_, both the valence and conduction bands are predominantly composed of I-p orbitals and Sn-s orbitals. The length of Sn-I bonds typically ranges from 3.04 Å to 3.20 Å, while that of Sn-Br ranges between 2.90 Å and 3.10 Å. The shorter distance observed between Sn-Br bonds compared to Sn-I bonds primarily arises from the fact that Br atoms possess one fewer outer shell electron than I atoms. Consequently, the reduction in atomic radius leads to a contraction of interatomic distances, with the combination of Sn and I being determinants of the bandgap size for CsSnI_3_ [33]. Overall, varying proportions of Cl atoms between 0 and 0.3 do not significantly impact the bandgap within the range of 1.5 to 2.0 eV; however, an appropriate ratio of Cl atoms contributes to stabilizing the bandgap. Discovering an improved element ratio to adjust the bandgap could enhance the response range of photovoltaic cells and improve the rate of solar spectrum response.

**Figure 7:**
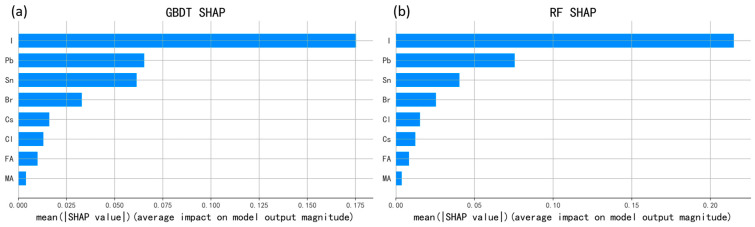
Ranking of feature importance generated by SHAP values of different algorithmic models: (**a**) using GBDT model; (**b**) using RF model.

While Figure 6 illustrates the influence of different ion ratios on the bandgap, it cannot precisely determine the degree to which each ion affects it. However, the SHAP interpretability machine learning framework proves highly effective in elucidating the main effects of specific features on target properties. Consequently, we selected GBDT and RF algorithm models with the best performance and differing algorithms. We then utilized SHAP to explicate the degree of influence of eight types of ions on ABX_3_ perovskite bandgap prediction, as depicted in Figure 7a,b. The bar graphs present all data samples with feature arrangement in descending order of SHAP mean absolute value, facilitating easy visualization of the most significant features affecting the bandgap. The length of each bar indicates the level of contribution of the feature to the target attribute. Despite discrepancies in algorithm models and their parameters, commonalities observed in Figure 7a,b reveal that B-site cations and X-site halides primarily influence the bandgap of perovskite. Among these components, I of X site has the greatest impact followed by Pb of B site, while A-site cations such as FA, Cs, and MA have minimal effects. Variations in ionic radii across different components induce lattice contraction or expansion. For example, if the radius of the A-site ion increases, the lattice expands, leading to a decrease in the bandgap. Similarly, doping X-site ions with larger halogen atomic radii results in a corresponding decrease in the bandgap [34], which is a crucial method for adjusting the bandgap in halide perovskites. The SHAP value interpretation precisely confirms the feasibility of this method. Therefore, from a physical standpoint, we can strive to achieve the desired bandgap value by adjusting the bond angle, bond length, or elements of B-X site ions. Considering that Sn atoms, beyond halogens, also influence the bandgap of inorganic perovskites, we can control it by either substituting Sn or coordinating with X atoms. This approach allows for the development of perovskite solar cells with enhanced performance, offering a promising avenue for future research.

**Figure 8:**
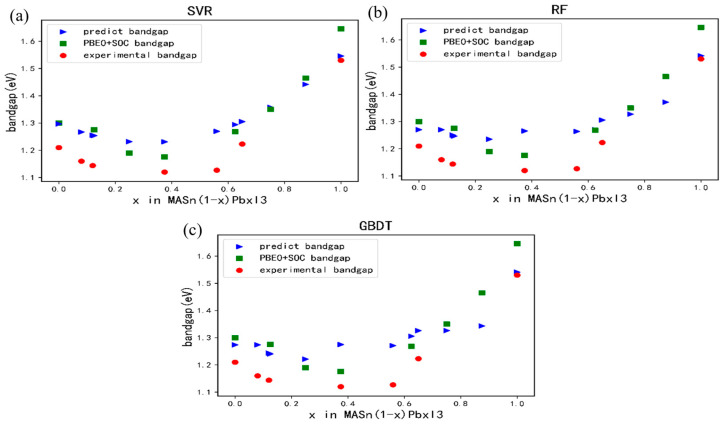
Experimental, theoretically calculated and predicted bandgap variations for MASn_(1−x)_Pb_x_I_3_ solid solutions of different compositions x. The blue triangles correspond to the model predicted bandgap values and the green squares represent the bandgap values calculated by the hybrid functional (PBE0) calculations including SOC. Red circles denote bandgap values from calculated experiments, (**a**) SVR model prediction; (**b**) RF model prediction; (**c**) GBDT model prediction.

In order to verify the accuracy of the model prediction, we compared the bandgap of the perovskite photovoltaic material MASn_1−x_Pb_x_I_3_ and analyzed the trend of the MASn_1−x_Pb_x_I_3_ material and the error of the model prediction by varying the different values of x. As shown in Figure 8, the blue triangles represent the bandgap values predicted by the model, the green squares represent the bandgap values calculated by the heterogeneous generalized function including SOC (PBE0 + SOC), and the red dots represent the experimentally calculated bandgap values [29]. We used the three models of SVR, RF, and GBDT to predict the MASn_1−x_Pb_x_I_3_ bandgap values, and the results show that the bandgap predicted by the model is basically consistent with this material, with a non-monotonic and highly non-linear bandgap evolution trend [29]. The predicted bandgap is somewhat overestimated compared to the experimental bandgap values, but the predictions are not significantly different compared to the bandgap values calculated from the hybridization generalization function (PBE0) including the SOC.

**Figure 9:**
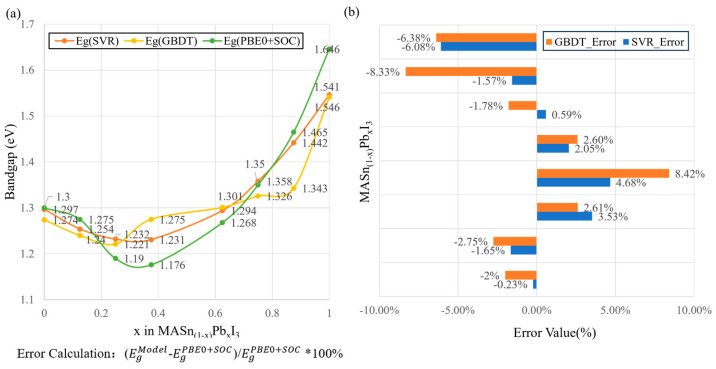
(**a**) MASn_(1−x)_Pb_x_I_3_ bandgap predictions for different components of x, including Eg (PBE0 + SOC), SVR and GBDT models. (**b**) Bandgap prediction errors of GBDT and SVR models.

It is noteworthy that among perovskite photovoltaic materials of this type, SVR demonstrates relatively superior prediction compared to GBDT and RF models. Particularly, SVR exhibits a smaller prediction error, especially within the bandgap range below 1.6 eV. The bandgap prediction errors for MASn_1−x_Pb_x_I_3_ materials with varying Pb atom compositions are depicted in Figure 9. As depicted in Figure 9a, the SVR predictions tend to smoothly approach the computed bandgap compared to the predictions by GBDT. Moreover, from Figure 9b, it is evident that the SVR prediction error is relatively smaller than that of GBDT. Therefore, from a practical standpoint, the SVR model may outperform GBDT in the narrow bandgap range, resulting in smaller relative errors in predicted values. However, it is important to note that considering the GBDT prediction model’s superior accuracy within the 1.4–2.0 eV bandgap range, its prediction capability remains excellent in larger bandgap ranges.

## 4. Conclusions

In summary, we compiled experimental data on the bandgaps of perovskite materials and developed multiple models using various machine learning algorithms. Leveraging the SHAP additive explanation model, we further elucidated the primary factors influencing the variation in bandgap of halide perovskites, yielding an overall feature ordering from the interpreted model. The results underscore the ensemble learning model’s ability to accurately predict perovskite bandgaps. Specifically, the GBDT model achieves an RMSE of 0.090 eV, a MAE of 0.053 eV, and an R^2^ of 93.11%. Moreover, the radial basis function kernel proves more adept at predicting narrow band perovskite. The bandgap reduction in perovskites stems from s-orbital hybridization induced by Sn atoms occupying the B site, while I ions at the X site facilitate bandgap adjustment within the optimal range, offering a practical approach for optimizing the bandgap of PSCs. Additionally, we successfully predicted the bandgaps of photovoltaic materials like MASn_1−x_Pb_x_I_3_. Our ML model accurately forecasts bandgaps across various ratios of metal and halide ions, aligning well with the non-monotonic and highly non-linear bandgap evolution trend observed in the material. This not only aids in predicting other binary systems but also provides guidance for ternary cationic perovskites, including MA, FA, and Cs.

Furthermore, the analysis of the model shows that the combination of that the decision tree model based on ensemble learning with SHAP provides an interpretable machine learning method, which helps to explore the underlying physical laws and chemical meanings behind the features, further promoting the development of a halide perovskite matrix system. The powerful implementation of the ensemble learning approach helps to purposefully discover novel ABX_3_ perovskites suitable for optoelectronic applications, significantly reducing the number of experimental errors in the laboratory and decreasing the amount of DFT theoretical calculations required. Therefore, this is consistent with the overall goal of materials informatics, accelerating the design and selection of materials, and thus promoting the development of the materials computation field.

## Figures and Tables

**Table 1:** The optimal hyperparameters of each model.

Model	Optimal Hyperparameters
SVR	‘C’:10,‘degree’:1,‘epsilon’:0.001,‘gamma’:0.05,‘kernel’:‘rbf’
KRR	alpha = 0.05, kernel = ‘rbf’, sigma = 10, gamma = sigma ** −2
GBDT	‘learning_rate’:0.01,‘loss’:‘ls’,‘max_depth’:6,‘max_features’:8, ‘min_samples_split’:8,‘n_estimators’:1000,‘random_state’:0,‘subsample’:0.9
RF	‘bootstrap’:True,‘max_depth’:None,‘max_features’:‘auto’, ‘min_samples_leaf’:1,‘min_samples_split’:2,‘n_estimators’:50
LightGBM	‘learning_rate’:0.171,‘max_depth’:5,‘n_estimators’:301,‘num_leaves’:55
Xgboost	‘colsample_bytree’:0.6,‘gamma’:0.1,‘learning_rate’:0.25, ‘max_depth’:6,‘min_child_weight’:2,‘n_estimators’:1000, ‘reg_alpha’:0.05,‘reg_lambda’:0.05,‘subsample’:0.7

**Table 2:** Scores of different machine learning models on train sets and test sets.

Model	Train Set			Test Set		
	MAE/(eV)	RMSE/(eV)	R^2^/(%)	MAE/(eV)	RMSE/(eV)	R^2^/(%)
KRR	0.063	0.089	91.4%	0.107	0.180	72.36%
SVR	0.050	0.088	91.6%	0.065	0.119	87.88%
RF	0.030	0.047	97.56%	0.063	0.100	91.48%
GBDT	0.014	0.025	99.30%	0.053	0.090	93.11%
LightGBM	0.030	0.047	97.64%	0.065	0.104	90.74%
Xgboost	0.049	0.073	94.23%	0.060	0.095	92.32%

## Data Availability

The raw data supporting the conclusions of this article will be made available by the authors on request.

## Supplementary Materials

The following supporting information can be downloaded at: https://www.mdpi.com/article/10.3390/ma17112686/s1, Figure S1: Scatter plot of 8-dimensional features; Table S1: Data Sources; Table S2: MASn_1−x_Pb_x_I_3_ data. References [23,24,25,26,35,36] are cited in the supplementary materials.
