# Supplementary material for: Employing the Interpretable Ensemble Learning Approach to Predict the Bandgaps of the Halide Perovskites
Source: Materials (Basel). 2024 Jun 2;17(11):2686. doi: 10.3390/ma17112686 (PMC11173945; doi:10.3390/ma17112686)
Supplement: Supplementary file 1 [file materials-17-02686-s001.zip › materials-2994748-Figure S1.pdf]

Supplementary materials

The ML models used in the experiments are:

Support Vector Regressor (SVR)[1] model is for linearly indistinguishable data to achieve data separability in a high-dimensional feature space. Due to the small number of features and normal number of samples, we use the support vector machine model based on radial kernel function.

Kernel Ridge Regression (KRR) is a model that uses a combination of kernel trick and ridge regression (linear least squares using L2 parametric regularization). It learns linear functions in a space formed by various kernels corresponding to nonlinear functions in the original space. This linear function in the kernel space is derived from ridge regression based on mean square error loss.

Both SVR and KRR use a radial kernel function based on Eq:

$$K(x_i, x_j) = \exp\left(-gamma \left\| x_i - x_j \right\|^2\right)$$

Where $x_i$andx_j are the 2 samples in the dataset, $K(x_i, x_j)$ is the similarity score between the samples, gamma is called the bandwidth parameter of the radial basis function. $|x_i - x_j|$ denotes the Euclidean distance between samples.

Random Forst (RF)[2] uses a modified CART decision tree as a weak learner, and obtains different training sample sets by resampling the samples, trains the learners separately on these new training sample sets, and finally combines the results of each one as the final learning result.

RF regression algorithm.

The input is the sample set D={(x_1,y_1),(x_2,y_2),...(x_m,y_m)}, and the number of weak classifier iterations T. The output is the final strong classifier f(x).

1. For t=1,2... ,T, there are .

1) random sampling of the training set for the tth time, a total of m acquisitions, to obtain a sampling set D_m containing m samples

2) The m-th decision tree model G_m(x) is trained with the sample set D_m. When training the nodes of the decision tree model, a part of the sample features are selected among all the sample features on the nodes, and an optimal feature is selected among these randomly selected part of the sample features to do the left and right subtree partitioning of the decision tree.

2. The arithmetic average of the regression results obtained by the T weak learners is the final model output.

Gradient Boosting Decision Tree (GBDT) is an additive model based on the idea of boosting integration, which uses a forward distribution algorithm for greedy learning during training, and each iteration learns a CART tree to fit the residuals between the predicted results of the previous (t-1) trees and the true values of the training samples. In short, the prediction accuracy of the model is continuously improved by building new models to compensate for the error of the previous model after training.

The principle of GBDT algorithm is as follows:

1. Initialization. Set the weights of all samples to equal values and establish an initial model as a benchmark model, which can be set as a simple mean or median. For example, establish a weak classifier F0(x)= $F0(x) = argmin_c \sum_{i=1}^{N} L(y_i, c)$, c is the average value.

2. Iterative training. In each round of iteration, the GBDT algorithm will first calculate the

residuals of each sample based on the prediction results of the current model. For the regression problem, the residual is the difference between the actual output value and the model prediction, and for the classification problem, the residual is the difference between the actual category of the sample and the model prediction category. Then, GBDT trains a new decision tree model to learn how to predict these residuals. For building M CART trees m=1,2,... .M.

1)For i=1,2,... ,N, calculate the response value (negative gradient of the loss function) corresponding to the mth tree:

$$r_{im} = -\left[\frac{\partial L(y_i, f(x_i))}{\partial f(x_i)}\right] f(x) = f_{m-1}(x)$$

2) For i = 1,2,... .N, the CART regression tree is used to fit the data to obtain the mth regression tree, whose corresponding leaf node region is R{m,j}, where j=1,2,... ,j{m}, and j{m} is the number of the mth regression leaf node.

3) For j_{m} leaf nodes region j=1,2,... ,j_{m},calculate the best-fit value

$$c_{m,j} = argmin_c = \sum_{x_i \in R_{m,j}} L(y_i, F_{m-1}(x_i) + c)$$

4) Update the strong learner f_m (x).

$$f_m(x) = f_{m-1}(x) + \alpha \sum_{j=1}^{j_m} c_{m,j} I(x \in R_{m,j})$$

3. Add a new model. The prediction results of the new model will be added to the output of the current model, making the prediction results of the model gradually converge to the true value. The output of each model can be weighted and summed to get the final model output.

4. Termination condition. When the accuracy of the model reaches a certain threshold or the number of iterations reaches a preset maximum, the algorithm stops iterating. Finally, the strong learner expression is obtained.

$$f_M(x) = F0(x) + \alpha \sum_{m=1}^{M} \sum_{j=1}^{J_m} c_{m,j} I(x \in R_{m,j})$$

The GBDT algorithm gradually improves the predictive power of the whole model by continuously training new decision tree models and accumulating their prediction results into the output of the current model. Compared with traditional decision tree algorithms, GBDT algorithm can reduce the risk of overfitting and has stronger robustness.

Extreme gradient boosting (Xgboost): Xgboost is based on the Boosting framework, which uses a forward optimization algorithm, i.e., it gradually builds the base model to optimize the approximation of the objective function from front to back. At the same time, a regularization component is added to reduce the loss function. In the optimization approach of the algorithm, the Xgboost loss function does a second-order Taylor expansion on the error part.

Light Gradient Boosting Machine (LightGBM)[3]: a gradient boosting framework based on learning algorithms, optimized to support parallelized learning based on GBDT, is a distributed and efficient Histogram-based decision tree algorithm. The advantage is to achieve parallelization and retain the accuracy while faster.

SHAP (SHapley Additive exPlanations, SHAP): An explanatory language developed from game theory in the field of machine learning, which can be used to explain the physical causes of machine learning results. What SHAP does is to quantify the contribution of each feature to the

predictions made by the model. It can visualize some of the relevant influence relationships, such as the influence of each feature vector on the target outcome.[4]

The Pearson correlation coefficient[5] is calculated as.

$$\rho = \frac{cov(X,Y)}{\sigma_X \sigma_Y} \qquad (1)$$

Where, there are,

$$cov(X,Y) = E(XY) - E(X)E(Y) \qquad (2)$$

In the above equation (1), the pearson formula ρ is defined as: the pearson correlation coefficient of two continuous variables (X,Y) is equal to the product of their covariances ("cov(X,Y)") divided by their respective standard deviations (σ_X σ_Y). Equation (2) is the formula for calculating the covariance[6].

The coefficient of determination is calculated as:

$$R^2 = \frac{SSR}{SST} = 1 - \frac{SSE}{SST} = 1 - \frac{\sum_i (y_i - f_i)^2}{\sum_i (y_i - \bar{y})^2} \qquad (3)$$

In the above equation (3), y_i is the actual value, f_i is the predicted value, and $\bar{y}$ is the average of the actual values. SST is the sum of squares, SSR is the sum of squares of regression, and SSE is the sum of squares of residuals.

The mean absolute error is the average of the absolute errors between the predicted and true values. It is calculated by Equation (4) as follows:

$$MAE = \frac{1}{n}\sum_{n=1}^{n} |y_i - \bar{y_i}| \qquad (4)$$

where n is the sample size, y_i is the actual value , and $\bar{y_i}$ is the predicted value.

The root mean square error is the square root of the mean of the squares of the errors between the predicted and actual values. The RMSE is used to illustrate the degree of dispersion of the sample. The smaller the RMSE, the better when doing nonlinear fitting. It is calculated by Equation (5) is as follows:

$$RMSE = \sqrt{\frac{1}{n}\sum_{n=1}^{n} (y_i - \bar{y_i})^2} \qquad (5)$$

where n is the sample size, y_i is the actual value , and $\bar{y_i}$ is the predicted value.

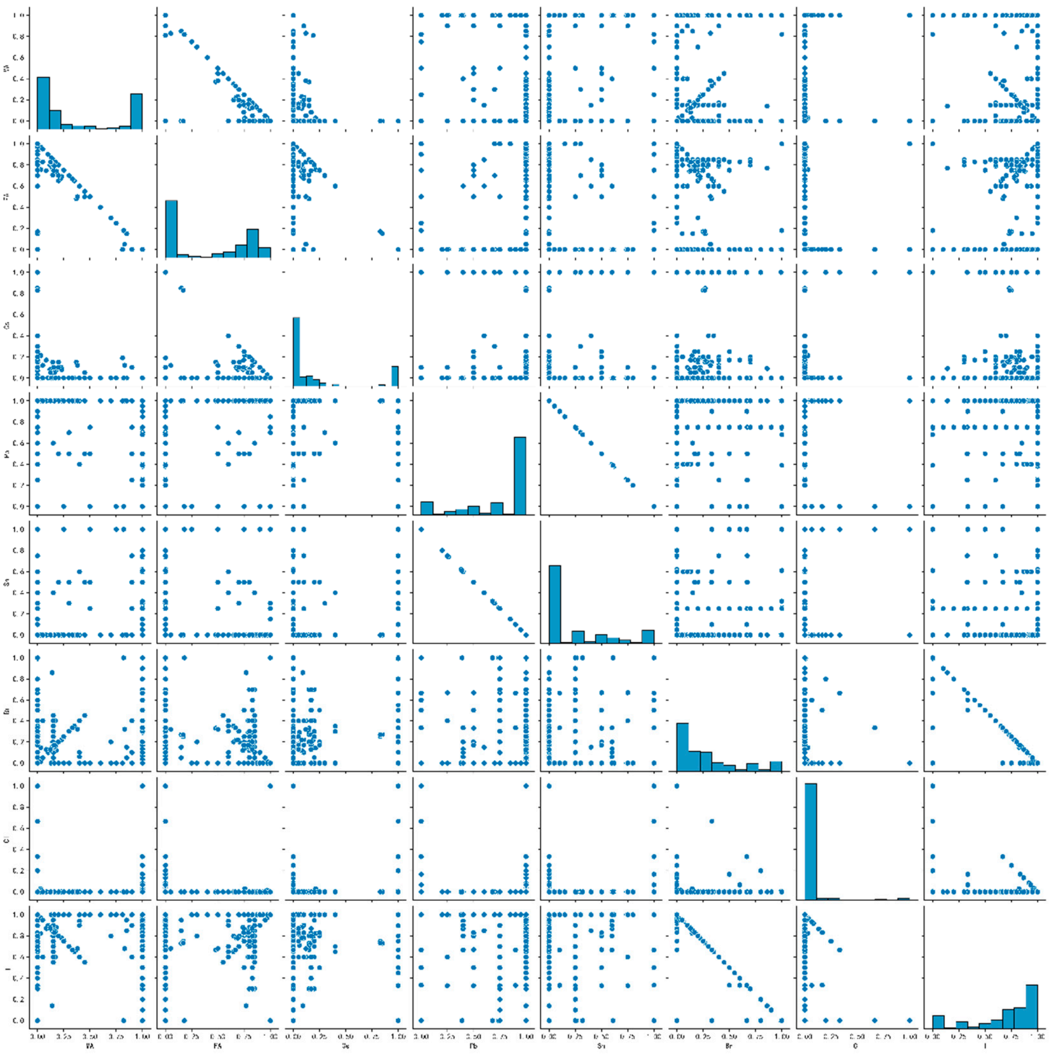

Fig.S1 Scatter plot of 8-dimensional features

Data Sources:

dataset.xlsx

$MASn_{1-x}Pb_xI_3$ data:

MASn1-xPbxI3.xlsx

Reference

[1] YERLIKAYA F A, BAHTIYAR S. Data poisoning attacks against machine learning algorithms[J]. Expert Syst. Appl. 2022,208: 118101

[2] GAO W, ZHI F X, ZHOU H. Towards convergence rate analysis of random forests for classification[J]. Artif. Intell. 2022, 313: 103788

[3] MANGALATHU S, JANGB H , HWANGC S H. Data-driven machine-learning-based seismic failure mode identification of reinforced concrete shear walls[J]. Eng. Struct. 2020, 208: 110331
[4] MANGALATHU S, HWANG S H, JEON J S. Failure mode and effects analysis of RC members based on machine-learning-based SHapley Additive exPlanations (SHAP) approach[J]. Eng. Struct. 2020: 219:110927
[5] Jebli I, Belouadha F Z, Kabbaj M I. Tilioua A Prediction of solar energy guided by pearson correlation using machine learning[J] Energy. 2021, 224: 120109
[6] Jain P, Jain S Can machine learning-based portfolios outperform traditional risk-based portfolios? the need to account for covariance misspecification[J] Risks 2019,7: 7
